# Chronic Patellar Dislocation Treated With Extensive Lateral Release and Vastus Medialis Obliquus Advancement: A Case Report

**DOI:** 10.1155/2024/5568998

**Published:** 2024-11-01

**Authors:** Shayne R. Kelly, Adam V. Daniel, Patrick A. Smith

**Affiliations:** ^1^Department of Orthopaedic Surgery, Missouri Orthopedic Institute, 1100 Virginia Ave., Columbia, Missouri 65201, USA; ^2^Department of Orthopaedic Surgery, Columbia Orthopedic Group, 1 S Keene St., Columbia, Missouri 65201, USA

## Abstract

The following case report demonstrates a case of a chronic irreducible patellar dislocation, age-indeterminate associated with a large medial patellar avulsion fracture that was treated with a vastus medialis obliquus advancement following an extensive lateral release. This case is the only known report of this kind in the literature. The patient is a 41-year-old Caucasian female who presented to the clinic with an age-indeterminate, chronically dislocated patella. She has a past medical history of hypertension and ischemic stroke 1 year prior to presentation, leading to expressive aphasia and lower extremity weakness in addition to patellar instability dating back to age 13. An unsuccessful patellofemoral reduction was performed at an outside clinic, and she was placed in a knee immobilizer and referred to our office. Clinically, the patient had limited knee range of motion with a fixed lateral patellar dislocation that was confirmed on imaging. This case report demonstrates a surgical reduction technique that can be utilized by orthopedic surgeons for chronic patellar dislocations that are not amenable to routine patellar instability surgeries due to the contraction of surrounding soft tissue, chronic bone abnormalities, and position of the chronic dislocation. An extensive lateral release followed by vastus medialis obliquus advancement was performed to center the patella within the trochlear groove and to allow for stable articulation throughout range of motion. The patient was able to regain painless, full range of motion of her knee postoperatively with patellar stability noted on both physical exam and radiographic imaging.

## 1. Introduction

Patellar instability is a condition that consists of patellar subluxation or dislocation episodes because of trauma, ligamentous laxity, or underlying risk factors like trochlear dysplasia, patella alta, and increased Q angle of the knee [1–4]. Patellar instability is common in young adults and may be treated conservatively following initial episodes but may require surgical intervention for recurrent patellar instability [3–5]. Surgical treatment options typically consist of medial patellofemoral ligament (MPFL) repair or reconstruction which is often combined with a tibial tubercle osteotomy (TTO) in the setting of an increased tibial tubercle-trochlear groove (TT-TG) distance [6–10]. Adjunct surgical options may include a lateral release and/or lengthening with potential trochleoplasty, which may be considered in severe or revision cases with trochlear dysplasia [4, 11].

In chronic patellar dislocations, a vastus medialis obliquus (VMO) advancement can be done to realign the patella [12, 13]. Given the chronic nature of this patient's patellar dislocation and abnormal anatomy, an MPFL reconstruction with a concomitant TTO was not performed. What makes this case unique is the presentation of the patient having a chronically dislocated patella for likely many months which remained laterally dislocated throughout all degrees of knee motion and was unable to be closed-reduced, even under anesthesia. Additionally, the presentation is complicated with her history of stroke affecting the involved extremity, along with a strong genetic component to her patellofemoral instability.

## 2. Case Presentation

This is a case of a 41-year-old Caucasian female who presented with an age-indeterminate patellar dislocation. She has a history of bilateral knee patellar instability that was treated conservatively beginning in her adolescent years. She has a past medical history including hypertension and a recent stroke that occurred 1 year prior to presentation caused by a thrombus that travelled through a patent foramen ovale. She subsequently developed moderate expressive aphasia and lower extremity weakness and was placed on Xarelto. The patient initially presented to a different orthopedic surgeon where an age-indeterminate patellar dislocation was diagnosed. The patient's family believed that the dislocation happened sometime after she had sustained the stroke when the patient flexed her knee, but they were only made aware of it when she complained of moderate anterior knee pain with ambulation. Ultimately, the patient was placed in a knee immobilizer, continued to weight bear with the assistance of a walker, and was subsequently referred to our office.

## 3. Clinical Findings

On physical examination, the patient was noted to have a severe 3+ effusion of her left knee. Her active range of motion (ROM) was limited from full extension to 40° of flexion which was limited by pain and guarding. Her patella could be easily palpated in a fixed laterally dislocated position. She had diffused medial parapatellar-type tenderness. She was stable on her ligamentous exam while a McMurray's test was unable to be performed due to her limited ROM and pain. She had marked quadriceps atonia and atrophy, and she was unable to perform a straight leg raise. She was neurovascularly intact to the extremity. Notably, her right patella demonstrated lateral hypermobility at 20° flexion with apprehension but was stable to lateral stress at 60° of flexion.

### 3.1. Diagnostic Assessment

**Figure 1:**
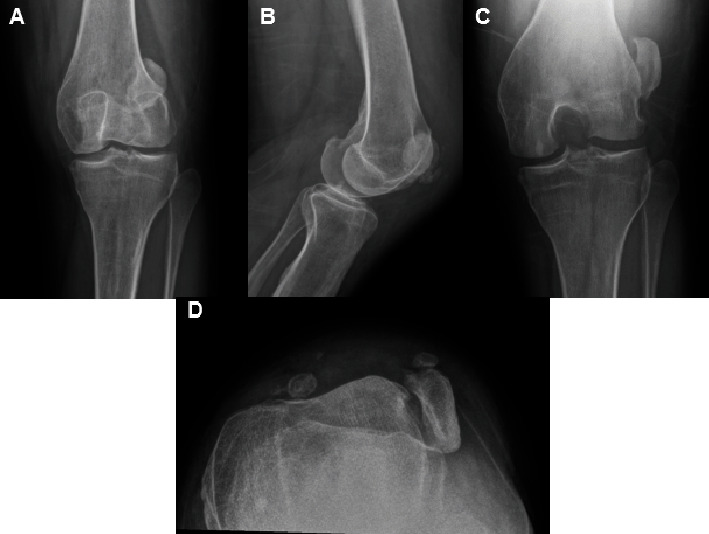
Preoperative radiographs. Preoperative radiographs of the patient's left knee done at an outside clinic. (A) Anteroposterior, (B) lateral, (C) tunnel, and (D) merchant radiographs demonstrating a complete lateral dislocation of the patient's left patella with associated large fracture fragment in dysplastic femoral trochlea with a chronic type avulsion calcification off the medial patella.

X-rays of the left knee were performed approximately a month and a half prior to her initial presentation to our clinic (Figure 1). These radiographs demonstrated a laterally dislocated patella with multiple displaced fracture fragments. It was not possible to determine patellar height due to the dislocated patella.

**Figure 2:**
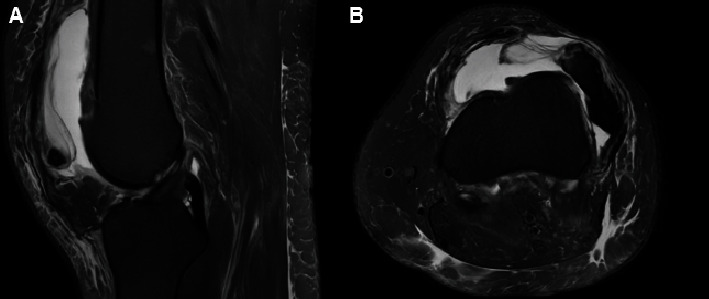
Preoperative MRI. T2 MRIs of the patients left knee. (A) Sagittal T2 MRI demonstrating a large joint effusion with a large fracture fragment located anterior to the femoral trochlea. (B) Axial T2 MRI demonstrating a large joint effusion with dysplastic femoral trochlea and a complete lateral dislocation of the patella.

MRI of the left knee was performed 1 month prior to her initial visit to our clinic (Figure 2). This study revealed a laterally dislocated patella with disruption of the MPFL ligament and medial retinaculum with an associated bony avulsion fracture. The femoral trochlea was noted to be dysplastic with edema and cartilage loss localized at the lateral trochlea. Her TT-TG distance was significantly elevated at 26.4 mm. Other structures of the knee appeared to be intact.

### 3.2. Therapeutic Intervention

Following the initial office visit, it was determined that this patient was to be treated surgically for patellar reduction and stabilization. We had a thorough discussion with the family discussing the risks, benefits, and alternatives of the surgery in addition to potential future complications and need for future surgeries. The original surgical plan was to perform an open reduction consisting of an extensive lateral release and lengthening, along with a proximal realignment consisting of MPFL reconstruction with an allograft. A TTO was also contemplated preoperatively. The goal of the surgery was to reduce and stabilize the chronically dislocated patella, to improve her ambulation and everyday function.

**Figure 3:**
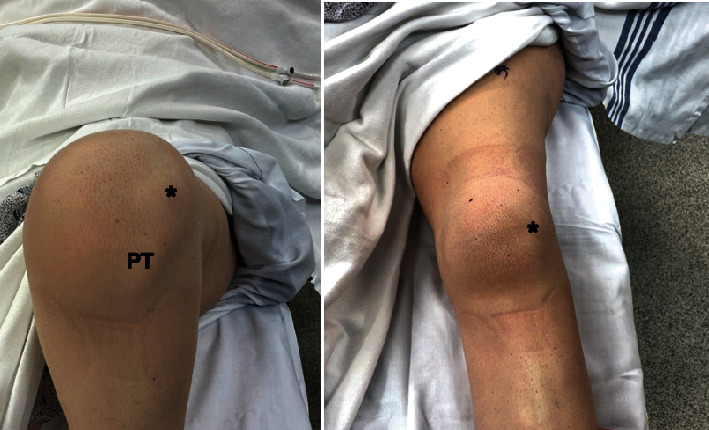
Intraoperative clinical exam photos. Chronically dislocated patella of the left knee (asterisk) more pronounced with knee flexion. PT, patellar tendon.

Intraoperative physical examination of the patient's left knee demonstrated no significant instability of the knee aside from the patella. Her patella was completely dislocated laterally and remained dislocated through knee ROM (Figure 3). Additionally, her right knee demonstrated lateral patellar dislocation to stress testing at both 20° and 60° of flexion but did not dislocate in the absence of patellar stress testing.

**Figure 4:**
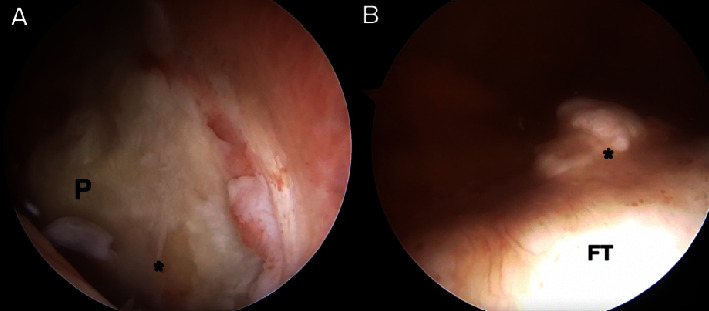
Arthroscopic view of patellofemoral joint. Arthroscopic view of the left knee from the anterolateral portal demonstrating a laterally dislocated patella (A) with chronic diffuse Grade 4 articular cartilage changes evident (asterisk) and a severely dysplastic femoral trochlea (B). P, patella; FT, femoral trochlea.

Diagnostic knee arthroscopy revealed a laterally dislocated patella within the lateral gutter and Grade 4 chondromalacia involving the entire central aspect of the patella as well as the lateral aspect of the femoral trochlea (Figures 4(A) and 4(B)). She was also noted to have a midbody radial tear of the lateral meniscus treated with a partial meniscectomy and a tibial spur anterior to the anterior cruciate ligament which was resected. The other structures of her knee remained intact.

Following arthroscopic evaluation of her knee, an open lateral release was performed by releasing the lateral retinaculum beginning at the joint line extending proximally alongside the dislocated patellar border. Despite a thorough release into the joint, the patella was still unable to be reduced. Further release included proximal dissection into the vastus lateralis muscle, down along the lateral edge of the patellar tendon, and ultimately through the fascia. Scarred fat pad was excised laterally and finally the patella was able to be reduced, but only with knee flexion at 70°. With higher degrees of flexion, the patella spontaneously dislocated.

Next, the medial retinaculum was incised beginning at the inferior pole of the patella extending up into the VMO proximally. The large avulsion calcification present in the medial retinacular tissue was located and excised. With the patella held centrally within the trochlea, the knee could only be brought to 90° of flexion before the tendon subluxated.

**Figure 5:**
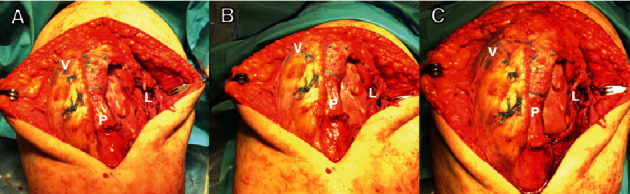
Intraoperative clinical photo. Clinical view of the patient's left knee following extensive lateral release with vastus medialis obliquus advancement with suture tapes. The patella remained within the femoral trochlea at (A) full extension, (B) 45° of flexion, and (C) 90° of flexion. P, patellar tendon; V, vastus medialis obliquus; L, released lateral retinaculum.

Following mobilization of the medial retinaculum and VMO off of the patella, a proximal realignment was done with VMO advancement. Initially, a strong suture (#2 FiberWire; Arthrex, Naples, FL, USA) was passed through the periosteal patellar tissue at the superomedial aspect of the patella and then back through the VMO. With the knee flexed at 90°, the VMO was then advanced distally on to the patella as this suture was tied. Following placement of this one VMO suture, the patella remained centered within the trochlea up to 110° of knee flexion. Then, with the knee flexed at 90°, three additional suture tapes (FiberTape; Arthrex, Naples, FL, USA) were placed within the periosteal patellar tissue and passed through the VMO in a horizontal mattress pattern for further reinforcement of the advancement. The knee was then taken from full extension to 110° of flexion with good tracking of the patella within the femoral trochlea (Figure 5). The knee was then closed in a standard fashion, sterile dressings were placed, and a hinged knee brace was the applied.

### 3.3. Postoperative Follow-Up and Outcomes

**Figure 6:**
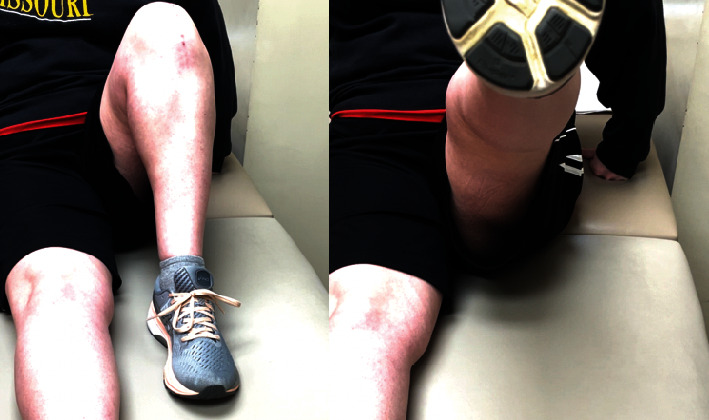
Postoperative clinical photo. In-office photograph of the patient at 6 months postoperative while maximally flexing her left knee to 115° of flexion and performing a straight leg raise with no extensor lag.

**Figure 7:**
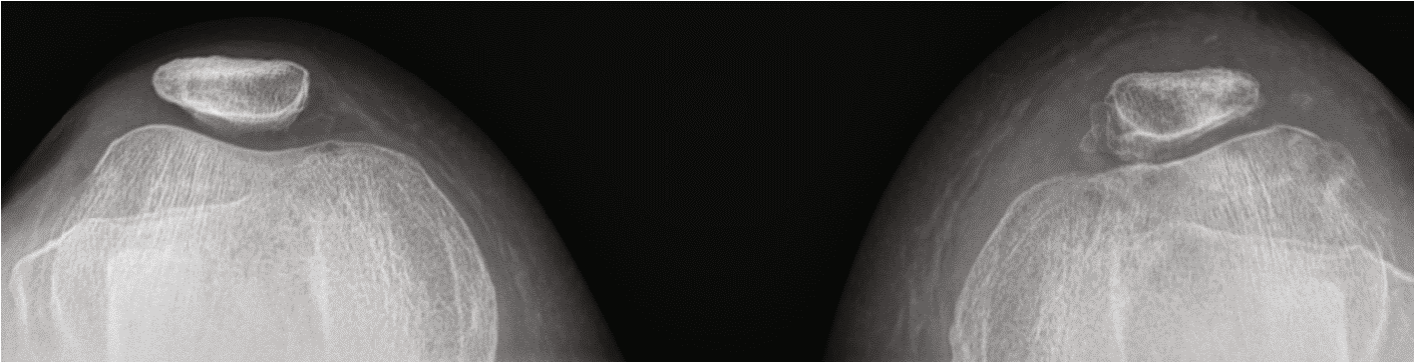
Merchant view of both knees 6 months postoperatively demonstrating centered patellae bilaterally.

Postoperatively, the patient was started on early ROM beginning in the recovery room with use of a continuous passive motion (CPM) machine (KinexCONNECT; Kinex Medical Company, Waukesha, WI, USA) along with physical therapy. She ambulated with partial weightbearing in a long-leg hinged knee brace locked in extension.  Table 1 summarizes the patient's physical examination findings at her postoperative visits. She was slow to gain quadriceps function postoperatively, but quadriceps function improved following her 6-week visit. At the 6-month follow-up, the patient had not had any episodes of instability, was able to actively flex her left knee to 115°, and was able perform a straight leg raise without lag (Figure 6). Her 6-month x-rays demonstrated centered patellae bilaterally on her merchant view (Figure 7).

Table 2 summarizes the patient's milestones and setbacks throughout the postoperative period. Overall, the patient has done remarkably well regarding her left knee. She can now painlessly ambulate on her own without her patellar stabilization brace.

## 4. Discussion

Patellar dislocations make up approximately 3% of all knee injuries, predominately affecting those in the second and third decades of life [4]. There is a paucity of literature describing the incidence and treatment of a chronically dislocated patella persistent at all degrees of knee flexion unable to be closed-reduced. Furthermore, there is limited literature surrounding the outcomes for chronic patellar dislocations and how they are surgically managed.

Typically, recurrent patellar dislocation is treated with a proximal realignment surgery such as an MPFL reconstruction utilizing allograft or autograft tendon tissue. In addition, distal realignment is oftentimes done with a TTO for patients with an elevated TT-TG distance of >20 mm and may even be considered for a TT-TG distance of 10–15 mm [6–10]. Unfortunately, this patient was not a good candidate for either of these procedures given her underlying bony pathology, quadriceps atrophy, soft tissue contracture, and global patellar articular cartilage damage. The MPFL reconstruction was originally planned; however, given the avulsion fracture and the overall thinness and small size of her patella potentially on a congenital basis, there was a major concern for iatrogenic patellar fracture during drilling and anchor placement—with graft inlay or onlay. A TTO was not performed due to concerns of disruption of the extensor mechanism in the setting of quadriceps atony and atrophy, concern of overloading her patellar cartilage damage with a TTO, and the likely need for future total knee arthroplasty that can be complicated with a TTO. Therefore, a VMO advancement was performed instead.

Additionally, it is important to mention that we suspect a genetic factor might have influenced the patient's knee condition. Her father, sister, and son have all encountered instances of patellar subluxation/dislocation, with varying degrees of severity among them. Specifically, her 19-year-old son has a documented history of fixed bilateral patellar dislocations for more than 3 years. While we acknowledge the potential contribution of the genetic component, the exact extent of its influence remains unknown, as does the question of whether treatment should be modified due to this genetic concern.

This case report was not without limitations. First, we did not obtain patient reported outcomes, so we were unable to subjectively assess the patient's progress pre- and postoperatively. Additionally, as there is limited data surrounding similar cases, we are unable to determine if this method of treatment truly was the best option for this patient. Although she has done well with improvement of her overall quality of life with her patella definitively reduced and stabilized, we do not have long-term follow-up, and she is at risk for future symptoms related to her patellar articular cartilage damage. Furthermore, due to the patient's expressive aphasia, it was difficult for us to obtain all the patient's thoughts and concerns throughout the entire process.

## 5. Conclusion

In conclusion, this case report presents a surgical reduction technique that orthopedic surgeons can use for chronic patellar dislocations that cannot be treated with standard patellar instability surgeries. The procedure describes an extensive lateral release followed by advancing the VMO to align the patella within the trochlear groove and ensure stability throughout full ROM. Following the surgery, the patient successfully regained pain-free knee ROM in addition to patellar stability noted on physical exam and radiographic imaging of the patella.

### 5.1. Clinical Message

Patellofemoral instability consists of a spectrum of disease that can be a difficult orthopedic problem to treat. When dealing with the most extreme chronic patellar dislocations, options of treatment may be limited and standard procedures likely will not suffice in obtaining stability and pain free ROM. Thus, it is important to develop other options such as a lateral release with VMO advancement to obtain the best outcomes possible from a difficult problem.

## Figures and Tables

**Table 1:** Postoperative physical examination findings at 2-week, 6-week, 3-month, and 6-month postop visits.

**Physical exam finding**	**2-week visit**	**6-week visit**	**3-month visit**	**6-month visit**
Effusion	2+	1+	Trace	None
Range of motion	0°–80°	0°–75°	0°–95°	0°–115°
Quadriceps tone	Poor	Poor	Good	Great
Straight leg raise	—	—	5° extensor lag	Without lag

**Table 2:** Postoperative protocol with milestones and events.

**Postoperative milestones and unanticipated events**	
Day 0	• Sent home with a CPM machine to facilitate early range of motion
Day 8	• Patient overflexed her knee by a couple of degrees while using her CPM with resultant increased discomfort • Recommended to continue with rest, ice, compression, and Tylenol for pain
Day 11	• Patient is at 82° of flexion with CPM • Made the transition from Xarelto to aspirin for DVT prophylaxis as per her cardiologist's recommendation • Prescribed additional Tramadol for pain management
Week 2	• Patient can ambulate with walker while her T-scope brace is locked in extension • Prescribed her first round of supervised PT • Continued use of CPM machine to better improve knee flexion
Week 3	• Patient began PT
Week 6	• Implemented blood flow restriction at PT to assist in quadriceps strengthening • Obtained postoperative radiograph of knee (merchant view only) • Patient fitted and supplied with a FreeRunner patella stabilizer brace
Month 3	• Fully transition from long-leg brace to smaller hinged patellar stabilization brace • Continued PT with focus on quadriceps and hip strengthening • Additional PT using the stationary bike for the promotion of knee flexion
Month 4	• Can now actively flex her knee to 110° in therapy • Can now perform straight leg raise without residual extensor lag • Graduated from supervised PT to home exercise program
Month 6	• Actively flex her knee to 115° • Can ambulate comfortably without brace

Abbreviations: CPM, continuous passive motion; DVT, deep vein thrombosis; PT, physical therapy.

## Data Availability

The authors have nothing to report.
